# Engineering a “detect and destroy” skin probiotic to combat methicillin-resistant *Staphylococcus aureus*

**DOI:** 10.1371/journal.pone.0276795

**Published:** 2022-12-15

**Authors:** Changhui Guan, Peter J. Larson, Elizabeth Fleming, Alexander P. Tikhonov, Sara Mootien, Trudy H. Grossman, Caroline Golino, Julia Oh

**Affiliations:** 1 The Jackson Laboratory for Genomic Medicine, Farmington, Connecticut, United States of America; 2 UCONN Health (University of Connecticut), Farmington, Connecticut, United States of America; 3 Azitra, Inc., Branford, Connecticut, United States of America; University of Mississippi Medical Center, UNITED STATES

## Abstract

The prevalence and virulence of pathogens such as methicillin-resistant *Staphylococcus (S*.*) aureus* (MRSA), which can cause recurrent skin infections, are of significant clinical concern. Prolonged antibiotic exposure to treat or decolonize *S*. *aureus* contributes to development of antibiotic resistance, as well as depletion of the microbiome, and its numerous beneficial functions. We hypothesized an engineered skin probiotic with the ability to selectively deliver antimicrobials only in the presence of the target organism could provide local bioremediation of pathogen colonization. We constructed a biosensing *S*. *epidermidis* capable of detecting the presence of *S*. *aureus* quorum sensing autoinducer peptide and producing lysostaphin in response. Here, we demonstrate *in vitro* activity of this biosensor and present and discuss challenges to deployment of this and other engineered topical skin probiotics.

## Introduction

The human skin plays host to diverse commensal microorganisms with a broad array of contributions to skin function, immune education, wound repair, and even protection against neoplasia [1]. Clinically, this is often overshadowed by its role as a reservoir for pathogens [2], prompting efforts to decolonize the skin with broad-spectrum topical antibiotics at the cost of more beneficial commensals. In particular, *Staphylococcus (S*.*) aureus* is a major human pathogen causing a wide range of skin and soft tissue infections, as well as a leading cause of bacteremia and device-related infections [3]. Methicillin resistant *S*. *aureus* (MRSA) is widely disseminated in both healthcare and community settings across 6 continents [4, 5]. An estimated 30% of the US general population is colonized by *S*. *aureus* and 2% with MRSA [6], with prevalence considerably higher (up to 60%) among healthcare workers [7]. The wide dissemination of MRSA constitutes a major loss in the war on antimicrobial resistance, limiting use of beta-lactams and their derivatives as first-line antibiotics for potential staphylococcal infections.

Skin colonization by *S*. *aureus* is a major public health challenge because it is considered to increase risk of infection and infection recurrence [8–10]. Decolonization is typically attempted with topical antiseptics (e.g., chlorohexidine, triclosan) and/or topical antibiotics (e.g., mupirocin), which have the risk of incomplete decolonization together with depletion of the local microbiota, which could further increase re-infection risk [11]. Recolonization rates are as high as 60% [11] following such procedures, and biocides such as triclosan have even been shown to promote *S*. *aureus* colonization [12]. Antibiotic-induced depletion of commensal microbiota, whose diversity has been shown to improve cutaneous health, resist pathogen colonization, and bolster host immune response to pathogens [2, 13], may be an added inducement for alternative strategies to manage MRSA colonization.

Following the model of recent development of engineered probiotics to combat enteric infections by *Pseudomonas* [14] and *Salmonella* [15] pathogens, we sought to develop a skin probiotic to combat MRSA colonization and growth on skin. Ideally, we envisioned a probiotic capable of specifically targeting MRSA and while sparing beneficial commensal microbiota. We engineered *S*. *epidermidis*, a ubiquitous skin commensal, to express anti-MRSA antimicrobials under control of a *S*. *aureus* quorum sensing regulator. This “detect and destroy” system intends to deploy of antimicrobials only where and when *S*. *aureus* is present, allowing for local bioremediation. Here, we present the *in vitro* activity and *in vivo* efficacy of our engineered skin probiotic and discuss ongoing challenges in skin probiotic development.

## Materials and methods

### Ethics approval and consent to participate

All mouse experiments were approved by the Jackson Laboratory Animal Care and Use Committee. Mice were euthanized via CO_2_ narcosis.

#### Strains, plasmids and reagents

All strains, plasmids and oligonucleotides (Table 1) used in this study are provided in the Supplementary Materials. *Escherichia coli* (*E*. *coli*) strains used for DNA cloning and sequencing were cultured in Lysogeny Broth (LB) (Difco Bacto) with or without 1.5% (w/v) agar. Plasmids were transformed into *E*. *coli* DH5α or *E*. *coli* dam-/dcm- chemically competent cells (New England Biolabs) according to the manufacturer’s protocol. Staphylococci were cultured in tryptic soy broth (TSB, BD Bacto) with or without 1.5% (w/v) agar. Antibiotics were added to the medium when needed: ampicillin (100 μg/mL), chloramphenicol (10–20 μg/mL), kanamycin (25–50 μg/mL) with concentrations depending on the host. Electrocompetent *S*. *epidermidis* ATCC12228 or Tü3298Δ*agr* was prepared according to previously published methods [16] with modifications; electroporation was performed with a Gene Pulser Xcell Electroporation Systems (Biorad), using manufacturer-provided protocol with the following modifications. 0.5–1 μg of plasmid DNA was incubated with competent cells for 30 min at room temperature. Electroporation was performed at 2,300 volts, 25 μF, 100 Ω with 2 mm cuvettes. Plasmid DNA was prepared using Plasmid DNA Miniprep or Midiprep Kit (Qiagen). Sanger sequencing services were provided by Eurofins or Genewiz. Precast 10–20% tricine protein gels (Novex) were used to perform SDS-PAGE. Primers for polymerase chain reaction (PCR) or sequencing were ordered from Eurofin Scientific or Integrated DNA Technologies (IDT). Cloning of all constructs was conducted in *E*. *coli* DH5α. Verified constructs were transformed into *E*. *coli dam-/dcm-* competent cells to generate intermediate plasmids for S. epidermidis transformation.

**Table 1:** Primer sets used for amplifying specific DNA fragments by PCR.

Amplified fragment	Size (kb)	Primer name	Sequence (5’ to 3’)
Type I agrCA	2.1	agrCagrA-BamHI-F	ATTTA**GGATCC**GAGAGTGTGATAGTAGGTGG
		agrCagrA-SalI-R	ATTTA**GTCGAC**GAATACGCCGTTAACTGA
P2P2 region	0.25	agr-P2P3-KpnI-F	AAACAGGTACCCAACTATTTTCCATCACATCTCT
		agr-P2P3-BamHI-R	AAATAGGATCCTTTTACACCACTCTCCTCAC
Type II agrCA	2.1	type2-agrC-BamHI-F	TTGAgGAtCCTAAAGTACCCGCTGA
		type2-agrA-SalI-R	TACAgTcGAcTACGCCGTTAACTGACT
Type III agrCA	2.1	type3-agrC-BamHI-F	TTGgGATccTTTATTGGATGAAGCTGAAGTACCAAAAG
		type3-agrA-SalI-R	AAGgTcgACAATTGAATACGCCGTTAACTGAC
Type IV agrCA	2.1	Type4-agrC-BamHI-F	TGTggaTcCATAATGGACGAAGTTGAAATACCT
		Type4-agrA-SalI-R	CAAgtcGAcTTGCATTTGAATACGCCGTTAACTG
Lysostaphin	1.5	Tet-ind-lystaph-F	GATGGTACCGCTTAAGGAGGATATTTTGAAGAAAACAAAAAACAATTATTATACGAGACC
		Tet-ind-lystaph-His-R	CCACCGCGGTGGCGGCCGCTTATCAATGGTGATGATGGTGGTGGGATCCTCCCTTTATAGTTCCCCAAAGAACACCT

#### Construction of staphylococcal biosensor reporters

*S*. *aureus* strains USA300-0114 (NR-46070, type I), NR-46204 (type II), NR-46081 (type III) and NR-45955 (type IV) were purchased from BEI Resources (www.beiresources.org). Primers for amplification of *agrCA* from the different *S*. *aureus* subtypes were designed using publicly available sequence data for these strains (GenBank Accession numbers: NR-46070-CAIHCM000000000.1, NR-46204-CAIHDX000000000.1, NR-46081- CP026066.1, NR-45955- CP026067.1). *S*. *aureus* genomic DNA was isolated with GenElute^™^ Bacterial Genomic DNA Kits from Sigma-Aldrich. *agrCA* and bidirectional promoter P2/P3 [17] were PCR amplified and ligated to plasmid pCN34 [18] sequentially. GFP gene was amplified from plasmid pCN57 [18] and ligated downstream of the P3 promoter in pCN34 and plasmid constructs were propagated into *E*. *coli* DH5a. For *S*. *epidermidis* genomic integration of the reporter constructs, the biosensor reporter cassette was cloned into temperature-sensitive *E*. *coli-S*. *aureus* shuttle vector pJB38 (gift from Jeffrey Bose), between two 1kb “arms” of the genomic sequences flanking the *agr* operon in *S*. *epidermidis* ATCC12228 for homologous recombination. Integration was performed according to methods previously published by Bose *et al*. [19].

#### Induction of biosensor reporters on plasmids in *S*. *epidermidis*

To test the specificity and cross-reaction of the *agrCA*-P2/P3-GFP reporter constructs, 1:10 dilutions of filter-sterilized supernatants from overnight cultures of the four *agr* subtypes of *S*. *aureus* (USA300/*agr* type I, NR46204/*agr* type II, NR46081/*agr* type III, NR45955/*agr* type IV) were co-incubated with *S*. *epidermidis* Tü3298Δ*agr* (1:100 dilution of fresh overnight cultures) harboring reporter plasmids of different subtypes in a total volume of 200 μL at 37°C for 5 hours with shaking in a 96-well plate (flat clear bottom and black sides, Cellstar). The cultures were centrifuged and washed twice with PBS and resuspended in 200 μL PBS, and the cell density (OD600) and fluorescence were read in BioTek Synergy2 plate reader.

#### Induction of chromosomal biosensor reporters in *S*. *epidermidis*

To test the sensitivity of each biosensor construct on the chromosome, genomic integrants of biosensor reporters (integrated at the *agr* locus, resulting in a knockout of the locus) for *agr* types I, II and III in *S*. *epidermidis* ATCC12228 were co-incubated with dilutions (1:1, 1:10, 1:100, and 1:1000) of filter-sterilized supernatants of *S*. *aureus* of their corresponding *agr* subtypes in the same way as above.

#### Construction and induction of antimicrobial biosensors

For antimicrobial peptide (AMP) expressing biosensor constructs, coding sequences for LL-37 (37 amino acids (AA)) and elafin [20] (61 AA) from human were synthesized by joint PCR with codons optimized for *Staphylococcus*. The gene for the bacteriocin hiracin (74 AA) from *Enterococcus hirae* [21] was PCR-synthesized without codon recoding. A gene encoding lysostaphin [22] (389 AA, *S*. *simulans*) was cloned from plasmid pDF8 in *Bacillus subtilis* BD170 (purchased from ATCC). The lysostaphin immunity gene was synthesized by joint PCR. To facilitate secretion of the antimicrobial products, a truncated signal peptide (MKFVKAIWPFVAVAIVFMFMSAA) derived from *S*. *epidermidis secA* secretion system [23] was cloned in front of the antimicrobial genes, except for the lysostaphin clone which contained an endogenous export motif [24]. A hexahistidine tag was added to the C-terminus of each AMP for purification.

The constructs were first cloned in plasmid pPL18 (Oh lab custom-made shuttle vector encoding kanamycin resistance, KanR) and the resulting plasmids (pPL18-AMPs) were transformed into *S*. *epidermidis* Tü3298 Δ*agr* for activity and inducibility test. Indicator strain *S*. *aureus* RN4220/pCN34 (plasmid pCN34 carries a KanR gene) was plated on TSB agar plates containing 25 μg/mL of kanamycin and different concentrations of anhydrotetracycline (ATc, 0.5, 1.0, 1.5, 2.0, 2.5, 3.0, 3.5 and 4.0 μM) [25]. Ten microliters of overnight cultures of *S*. *epidermidis* Tü3298Δ*agr* harboring pPL18-AMPs were spotted on top and the plates were incubated at 37°C overnight, then at room temperature for two more days for activity scoring.

To verify the expression and secretion of the antimicrobial peptides, one hundred milliliters of the bacterial cultures of *S*. *epidermidis* Tü3298Δ*agr* containing pPL18-AMPs were induced at OD600 ~1.0 with 1.0 μM of ATc at 30°C for 4 hours. Batch purification of AMPs was carried out as follows: Two percent (v/v) of 1 M Tris-HCl, pH 8.0, and 10% (v/v) Ni-NTA resin (Qiagen) were added to the cell-free supernatant which was stirred at 4°C overnight to facilitate resin-peptide binding. The resin was collected in centrifuge tubes the next day and washed with washing buffer (20 mM Tris, 500 mM NaCl, 20 mM imidazole, pH 8.0). The bound peptide was eluted with elution buffer (20 mM Tris, 500 mM NaCl, 250 mM imidazole, pH 8.0). The eluate was dialyzed against phosphate buffered saline (PBS) and concentrated with Amicon spin columns (pore size 3 KD) with phosphate buffered saline (PBS). The samples were then subjected to SDS-PAGE analysis.

#### Integration and induction of biosensor antimicrobial peptide (AMP) constructs

The antimicrobial biosensor constructs for *agr* type I, II, and III expressing lysostaphin were created by replacing the GFP gene with lysostaphin and its immunity genes in appropriate biosensor reporter constructs in the integrative plasmid pJB38. They were integrated via homologous recombination into the genome of *S*. *epidermidis* Tü3298 Δ*agr* at the *agr* locus. Integration and DNA sequences were verified by sequencing the genomic PCR products. *S*. *aureus* of four *agr* types were plated on TSB agar plates and 10 μL containing 10^4^ and 10^6^ CFU of each of two individual integrants for each *agr* type were spotted on top. Parent strain *S*. *epidermidis* Tü3298 Δ*agr* was included as control. The plates were incubated at 37°C overnight, then at room temperature for two more days for activity scoring.

#### Mouse trials

Germ-free C57BL6/J mice were purchased from The Jackson Laboratory, Bar Harbor, ME and maintained at room temperature in a germ-free facility at UConn Health. To prepare bacterial inocula, overnight cultures (OD600 for all ~13) of *S*. *aureus* USA300, *S*. *epidermidis* Tü3298Δ*agr* (parent) and *S*. *epidermidis* type I biosensor producing lysostaphin (biosensor) were centrifuged and the pellets were washed twice with sterile H_2_O. Groups of five mice were used, except where otherwise noted. The pellets of each individual strain or *S*. *epidermidis-S*.*aureus* mixtures were suspended in ~ 1.5 mL of Vaseline brand petroleum jelly [26], and applied as described below. For CFU quantitation, samples were collected by PurFlock Ultra buccal swabs (Puritan Medical Products) moistened in buffered solution (50 mM Tris-HCl, 1 mM EDTA and 0.5% Tween-20, pH 8.0), swabbing both ears for each sampling, and CFU quantitation was performed by spread plating onto mannitol salt agar (MSA) to differentiate *S*. *aureus* from *S*. *epidermidis* colonies. After incubation at 37°C overnight, colonies were counted, and identification of select colonizes were verified by MALDI-TOF. Colony counts over 2,500 were estimated.

For experiments examining growth dynamics after co-colonization, mice were inoculated singly with ~10^9^ CFU of *S*. *aureus* USA300, *S*. *epidermidis* parent, or biosensor, or a mixture of *S*. *aureus*/*S*. *epidermidis* parent or biosensor (~10^7^/10^9^ CFUs, respectively). We found that colonization with a single strain was robust and relatively stable over the course of the experiment, with no significant differences between the three strains (Fig 3A). Each mouse was inoculated on both sides of both ears with the appropriate bacteria-petroleum jelly suspension on days 0, 2 and 4, described below. Beginning on day 2 and every other day through up to day 10, staphylococcal colonization was quantified as described above, prior to bacterial application.

For experiments examining growth dynamics after precolonization by *S*. *epidermidis*, mice were first colonized with ~10^9^ CFUs parent or biosensor on day 0. On day 2, the mixture of *S*. *aureus*/*S*. *epidermidis* parent or biosensor (~10^7^/10^9^ CFUs) was applied. For one trial, an additional dose of ~10^9^ CFUs parent or biosensor was applied on day 4, with negligible effect. Beginning on day 2 and every other day through up to day 10, staphylococcal colonization was quantified as described above prior to bacterial application.

For wound infection assays, germ-free mice were shaved, and then 4mm punch biopsies were performed to create wounds in the dorsal skin. A mixture of *S*. *aureus*/*S*. *epidermidis* (~10^5^/10^8^ CFU) in petroleum jelly was then inoculated into the wound. After 48 hours, a 1.5 cm x 1.5 cm area surrounding the wound was excised and harvested in PBS buffer. Bacteria were dissociated from skin in the PBS suspension by applying motorized Pellet Pestle (Fisher) disruption for 10 seconds, and dilutions were spread on MSA plates for CFU quantitation. n = 5 mice per group in initial trial, n = 12 for repeat trial.

#### Data analysis and statistics

**Fig 1:**
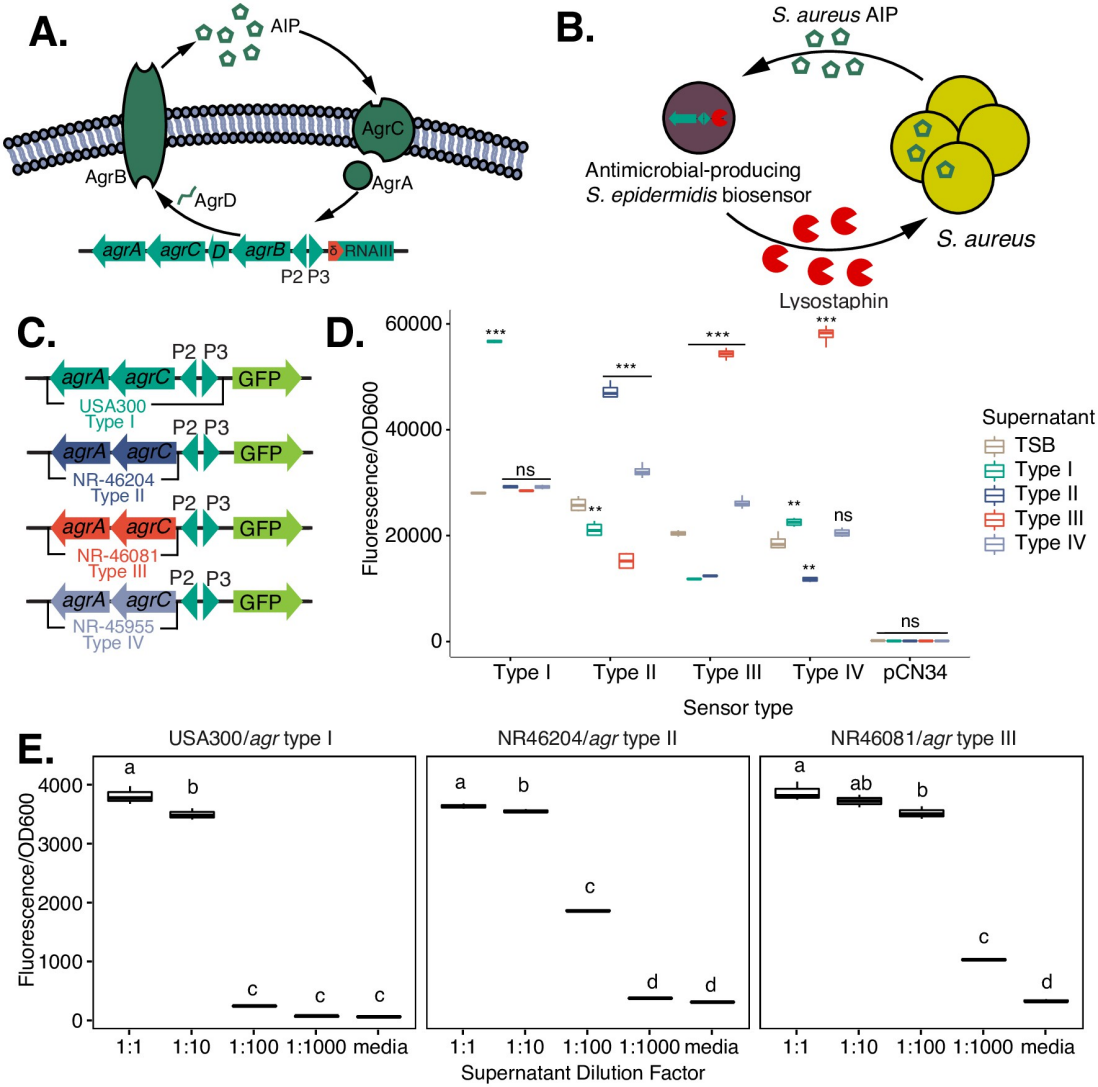
Biosensor design. **A)** Illustration of the staphylococcal *agr* quorum-sensing circuit. AgrD pro-peptide is modified and exported by AgrB as an auto-inducer peptide (AIP). AIP stimulates two component sensor AgrCA, which upregulates transcription of the P2/P3 promoter, upregulating *agr* component and RNAIII expression. **B)** Concept illustration of a *S*. *epidermidis* “detect and destroy” probiotic biosensor. The sensing component from *S*. *aureus’* quorum sensing circuit in A) (*agrA* and *agrC)* are introduced into an *agr* mutant *S*. *epidermidis* strain. Binding of AgrA to the P2/P3 promoter controlling expression of a *S*. *aureus*-targeting antimicrobial then results in density-dependent production of the antimicrobial of choice, here, lysostaphin. **C)** Staphylococcal biosensor circuit design for validation. *agrCA* sensor genes were cloned from *S*. *aureus agr* groups I-IV and placed downstream of promoter P2, with GFP under control of P3. **D)** Biosensor induction by different AIPs from different *S*. *aureus agr* types, testing the efficacy and the cross-reactivity of a plasmid-borne biosensor. Supernatants of overnight *S*. *aureus* cultures from each *agr* type were filter-sterilized, diluted 1:10 in TSB and co-incubated with *S*. *epidermidis* strains expressing the corresponding *agr* biosensor reporter constructs (sensor). Control was empty vector (pCN34). Bidirectional T-test compared to TSB control group, *: p ≤ 0.05, **: p ≤ 0.01, ***: p ≤ 0.001, n = 2 replicates. **E)** Testing efficacy and sensitivity of biosensor induction by the matched *agr* type AIP, using a biosensor integrated into the *S*. *epidermidis* genome. Given low cross-reactivity from D), we tested each biosensor with its matching *agr* type in a dilution series to test sensitivity. Biosensors were co-incubated with dilutions of supernatant from *S*. *aureus* strains of the matching *agr* type. Media was used as control. Significance between groups determined within each panel by ANOVA with post-hoc Tukey HSD test, p < 0.05, n = 3 replicates. Groups that do not share the same letter (a, b, c, or d) are significantly different in post-hoc multiple comparisons test.

All data analysis was conducted in R (v4.0.2). Statistical significance in this study was attributed to tests wherein p < 0.05. Induction or suppression of plasmid biosensor fluorescent reporter by different AIPs (e.g., Fig 1D) was determined with a bidirectional T-test comparing to fluorescence of the TSB treatment group for the media sensor. Induction of integrated reporter constructs (e.g., Fig 1E) was assessed using an analysis of variance (ANOVA) with a post-hoc Tukey HSD test to assign groups. Linear regressions were performed using the Spearman method. Comparison of CFU counts over time between groups in mouse trials (Fig 3B-3E) was performed with analysis of co-variance (ANCOVA). Comparison of CFU burden in wound assays (Fig 3F-3G) was performed with a bidirectional T-test.

## Results

### Biosensor design

First, we selected *S*. *epidermidis* as the background for our engineered probiotic because it is a ubiquitous skin commensal [27]. Its widespread ability to colonize skin was desirable as we hypothesized that it might be able to then protect against MRSA anywhere on the body. Although relatively challenging to genetically modify, it is still among the most tractable of skin commensals [19].

Second, we desired that it would secrete a narrow-spectrum but anti-MRSA effector, and only do so in the presence of *S*. *aureus*. We reasoned that this was important because constitutive secretion of potentially toxic compounds could have undesired effects on skin and surrounding microbiome as well as potentially promote acquisition of resistance. Our design of biosensing co-opts *S*. *aureus* quorum sensing, a density-dependent mechanism by which many pathogens regulate virulence on a population scale by secreting chemicals called autoinducer peptides (AIPs) that accumulate extracellularly until a threshold concentration is achieved to trigger an alternative transcriptional program [28]. *S*. *aureus* AIP, which is species-specific [17], presumably could be detected by *a S*. *epidermidis* strain that possesses the corresponding signal transduction cascade.

In *S*. *aureus*, quorum sensing is controlled by the *agr* (accessory gene regulator) circuit [28]. Precursor peptide AgrD is modified and secreted by AgrB as an auto-inducer peptide (AIP). AgrC and AgrA form a two-component signaling system which upregulates the transcription of promoters P2 and P3 in response to AIP binding [17]. For expression in *S*. *epidermidis*, we cloned the *agrCA* sensor and P2/P3 promoter from MRSA strain USA300, assembled them into a staphylococcal shuttle vector such that *agrCA* remained under P2, and placed a GFP reporter gene downstream of P3.

In addition, because four different *agr* groups have been identified in *S*. *aureus*, each representing different phylogenetic groups [29], we developed sensors targeting all four groups. *agrA* and P2/P3 are highly conserved (98–100% nt identity) between *agr* groups, so we built derivative constructs by substituting the USA300 *agr* type I *agrCA* with *agrCA* cloned from representative strains of type II (derived from type II *S*. *aureus* strain NR46204), type III (NR46081), and type IV (NR45955). Finally, we expressed these plasmid constructs in *S*. *epidermidis* strain Tü3298Δ*agr* [30]. An *agr* deletion mutant was used to avoid recombination events between high sequence identity genes, induction of the reporter gene by endogenous *agr* signaling, and to circumvent potential cross-inhibition between *S*. *epidermidis* and *S*. *aureus agr* circuits [17, 31], which could cause this biosensor to block itself. The overall design is shown in Fig 1.

To test the activation of these prototype biosensors, we examined the response to AIP from its own group as well as cross-reactivity between groups (Fig 1D). Here, we used a plasmid-borne biosensor, incubating clones of each biosensor type with a 1:10 dilution of culture supernatant from self or other *agr* types. As expected, supernatant from other *agr* subtypes had variable effect on biosensor induction. The most potent induction (>2-fold increase in fluorescence over media control) typically occurred with its matched type (e.g., type I supernatant with type I biosensor), and cross-reactivity of the type I and type II biosensors was low. Interestingly, the type III biosensor had similar strong activation from both type III and type IV supernatant, which may be consistent with the consideration of *agr* type IV as a rare transitional genotype, having properties that are similar to multiple *agr* types [32]. Finally, the type IV biosensor had low activation by any supernatant including itself and had cross-reactivity with the type III strain. Because type II and type III *agr* biosensors were able to detect type IV AIP more effectively than the type IV biosensor itself, and type IV *agr* strains have also been shown to have weak cross reactivity with *agr* type I AIP [32], we excluded the type IV construct from subsequent experiments. We note that the observed differences in cross-interactions were not entirely consistent with published interactions between *agr* groups [29, 32], and could arise from the heterologous expression setting, or other elements of the supernatant such as bacteriocins, extracellular enzymes, or pH differences.

We noted that our plasmid-borne biosensor had relatively high background GFP expression (Fig 1D), likely due to low constitutive expression from the P2/P3 promoter combined with the moderate copy number of the plasmid. Given that our ultimate goal is to express toxins or antimicrobial peptides (AMPs), it would be ideal to have a system with lower basal expression. We hypothesized that copy number could contribute to high background expression (though vector pCN34 is characterized as a low copy staphylococcal plasmid [18]), so we integrated the type I-III biosensor constructs into the chromosome at the residual *agr* locus and tested activation and cross-reactivity as described above. We observed significantly reduced background GFP expression and a dose-dependent induction, with induction occurring most potently at 1:1 or 1:10 dilutions, albeit with a reduction in overall expression (Fig 1E). While this is a tradeoff to minimizing background expression that may reduce biosensor efficacy, we deemed it necessary, and it had the added benefit that no agents were subsequently needed to maintain plasmid-borne transgenes, which could present additional challenges to deploy *in vivo*.

Having confirmed that gene expression in *S*. *epidermidis* could be controlled by *S*. *aureus* quorum signaling *in vitro*, we next sought to determine what antimicrobials might be a feasible output. Our major considerations were 1) effective inhibition of clinically important *S*. *aureus* strains such as USA300, 2) potential to encode self-immunity in the *S*. *epidermidis* biosensor, and 3) feasibility of heterologous expression (i.e., larger constructs might be more difficult to deploy). Our initial trials included epidermin, a native, plasmid-borne *S*. *epidermidis* lantibiotic with a broad spectrum of activity that is readily expressed heterologously with built-in self-immunity [33]. However, it had no inhibitory activity against most *S*. *aureus* strains (not shown). We then attempted dual plasmid systems to pre-express resistance cassettes to lantibiotics gallidermin [33] and mersacidin [34], then introduce the synthesis genes in a second vector. This approach had limited success, likely due to the size of the operon or transgene toxicity. Given these challenges, we adjusted our strategy to focus on small peptides or proteins that could be produced with a single heterologous gene. We compiled a library of ~200 bacteriocin genes/peptides from the literature and relevant databases (e.g., Uniprot at https://www.uniprot.org/ [35], Bactibase at http://bactibase.hammamilab.org/ [36]) and evaluated these for source, size, self-immunity, post-translational modifications required, requirement for transporters, potential host resistance, and activity against *S*. *aureus*. We identified several strong candidates, including human AMPs LL-37 [37] and elafin [20], *E*. *hirae* bacteriocin hiracin [21], and *S*. *simulans* bacteriolytic enzyme lysostaphin [22]. The former two were of interest given their size (<10 kDa) and potential tractability, despite that they would likely be toxic to the expression host, and the latter two were of interest for their size (8 kDa and 42 kDa, respectively) and their potential for encoding expression host resistance. Each had demonstrated activity against *S*. *aureus* with no or less potency against *S*. *epidermidis* than had been previously reported, except for lysostaphin, for which we introduced a facilitator gene for resistance.

**Fig 2:**
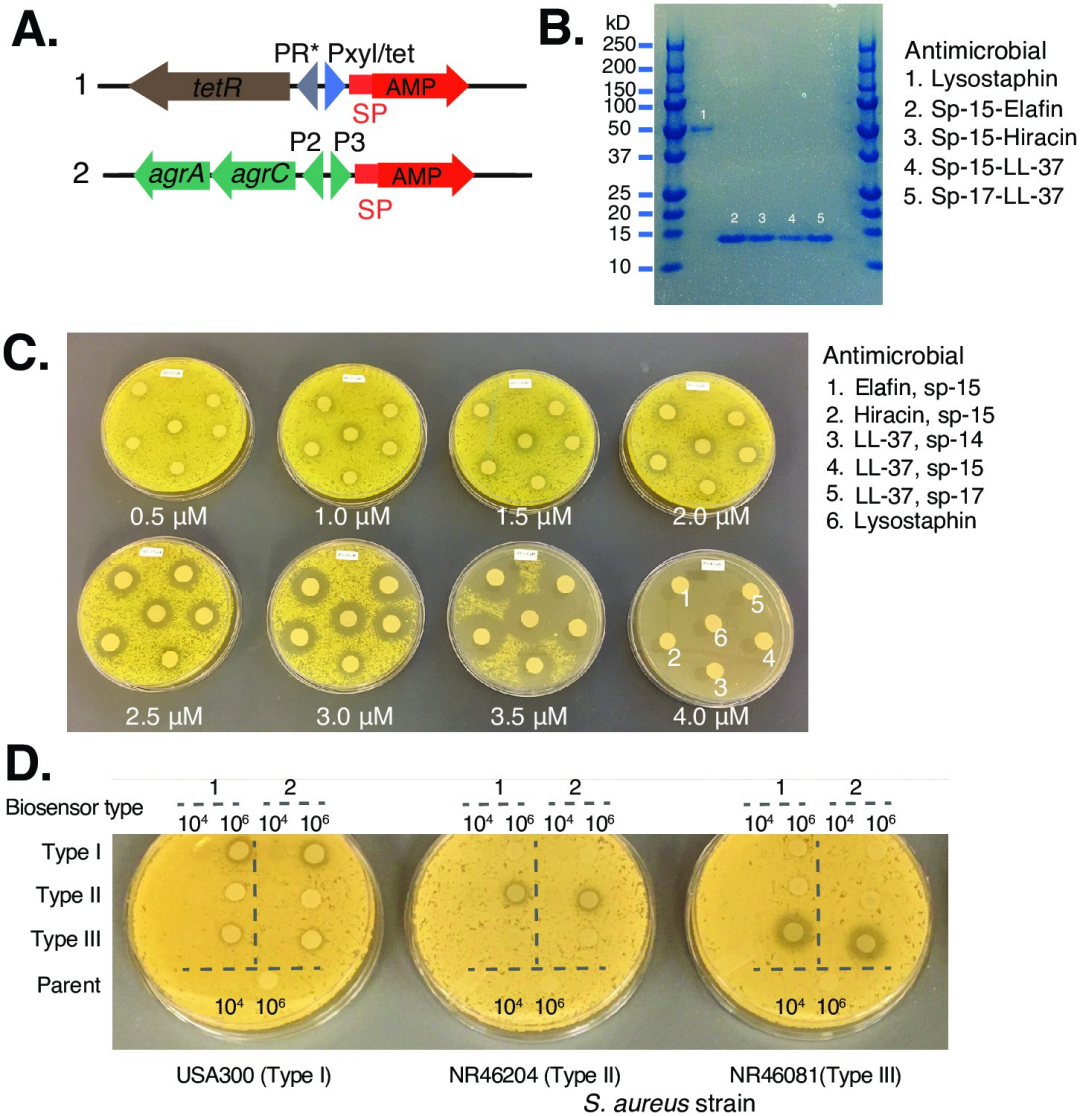
*In vitro* efficacy of *S*. *epidermidis* producing different antimicrobials. **A)** Concept of design to express antimicrobial peptide (AMP) genes under the biosensor. AMPs are tagged for export with a signal peptide (SP) in *S*. *epidermidis* under control of either (1) tetracycline inducible promoter *(Pxyl/tet)* for testing under tight inducible control, or (2) *agr* P2P3 biosensor. **B)** Pull-down SDS-PAGE verifying AMPs elafin, LL-37, hiracin, and lysostaphin secretion from *S*. *epidermidis*. Several different signaling peptides (e.g., SP-14, SP-15, SP-17) were tested for LL-37. The endogenous signal peptide for lysostaphin was used. **C)** *S*. *epidermidis* expressing AMPs under the tet-inducible promoter demonstrate strong growth inhibition of *S*. *aureus* in an overlay assay. Concentration of anhydrotetracycline (ATc) is shown and is dose-dependent. **D)** *In vitro* activity and specificity of lysostaphin-expressing *agr* type I, II, and III biosensors integrated into Tü3298Δ*agr* genome. Parent strain included as control. Two biosensor integrant clones (i.e., biological replicates “1” and “2”) were tested at initial doses of 10^4^ and 10^6^ CFUs of *S*. *epidermidis* spotted on for each overlay assay.

We first tested if these four candidates could be expressed heterologously in *S*. *epidermidis*. Because of potential toxicity, we tested them under a tightly controlled inducible tetR/Pxyl/tet promoter system [25] (Fig 2A). Self-immunity genes were included for hiracin and lysostaphin to prevent self-killing. To identify potential effective secretion signal tags for the antimicrobials, we screened a Gram + Sec-type signal peptide (SP) library [23], cloning them onto the 5’ end of LL-37 in *E*. *coli* as our test case. Out of 11 unique SPs identified in *E*. *coli* hosts, we were able to transform 9 into Tü3298Δ*agr*. Of these, we performed a growth curve inhibition assay with the assumption that the more effectively LL-37 is secreted upon anhydrous tetracycline (ATc) induction, there would be less growth defect from the intracellular accumulation of toxic product. Two clones showed no changes in doubling time (SP-15 and SP-17) and one clone showed a slight slowing in doubling time (SP-14) after induction. All three were selected for proof-of-principle testing with LL-37 (Fig 2B), and SP-15 was used for the remaining candidates, except for lysostaphin, for which we used the endogenous signal peptide [24].

We verified the expression and secretion of the antimicrobials by purification of the peptides from supernatant with a Ni-NTA pulldown assay and SDS-PAGE (Fig 2B). Then, to ascertain that the secreted peptides were active against *S*. *aureus*, isolates were grown in an *S*. *aureus* agar overlay assay with increasing concentrations of ATc for antimicrobial induction. Activity was scored by zone of inhibition (Fig 2C), with all antimicrobial-producing isolates showing potent activity against *S*. *aureus* with a linear dose-response to ATc. We noted that the activity of LL-37 with SP-14 was lower than the same versions with other signal peptides, likely due to a different sequence composition in the signal peptide, consistent with the observed growth defect. For example, with 3.0 μM of ATc, the inhibition zone of LL-37/SP-14 had a diameter of 14 mm, while LL-37 with other SPs had a diameter of 21 mm. Under the tested conditions, all AMPs showed dose-dependency: with ATc at 2.5, 3.0, and 3.5 μM, the diameters of the inhibition zones were consistent (with the exception of LL-37/SP-14) at 17, 21 and 28 mm, respectively. Taken together, we concluded that *S*. *epidermidis* was an effective producer of *S*. *aureus* antimicrobials irrespective of previously reported activity against *S*. *epidermidis* itself (i.e., LL-37 [37]).

Given comparable efficacy between these select antimicrobials, we proceeded with lysostaphin as our proof-of-principle in the biosensor construct, as it has the narrowest spectrum of activity given that its substrate is a staphylococcal peptidoglycan, and genes encoding resistance can also be cloned into the biosensor host. In addition, its minimum inhibitory concentration (MIC) as well as its bacteriocidal dynamics against *S*. *aureus* is well characterized (killing *S*. *aureus* within minutes and MIC at which 90% of the strains are inhibited [MIC_90_], 0.001 to 0.064 μg/ml [22]). We placed the lysostaphin-encoding locus under the type I-III biosensors and integrated the resulting constructs plus resistance gene into the Tü3298Δ*agr* genome (Fig 2A). We sought to examine its activity in an agar overlay assay, which differs from the *in vitro* liquid assay as interactions of the biosensor strain with *S*. *aureus* is spatially defined. When incubated with agar overlays of *S*. *aureus* strains from each *agr* group, we observed corresponding zones of inhibition for each target group (Fig 2D). Activities for *agr* groups I, II, and III were *agr* group specific, with negligible cross-activation (Fig 2D). These results confirmed that *S*. *epidermidis* could be engineered to “detect and destroy” *S*. *aureus* in an *agr* group-specific manner *in vitro*, excluding *agr* group IV, which we found had similar cross-reactivity in this assay as well.

**Fig 3:**
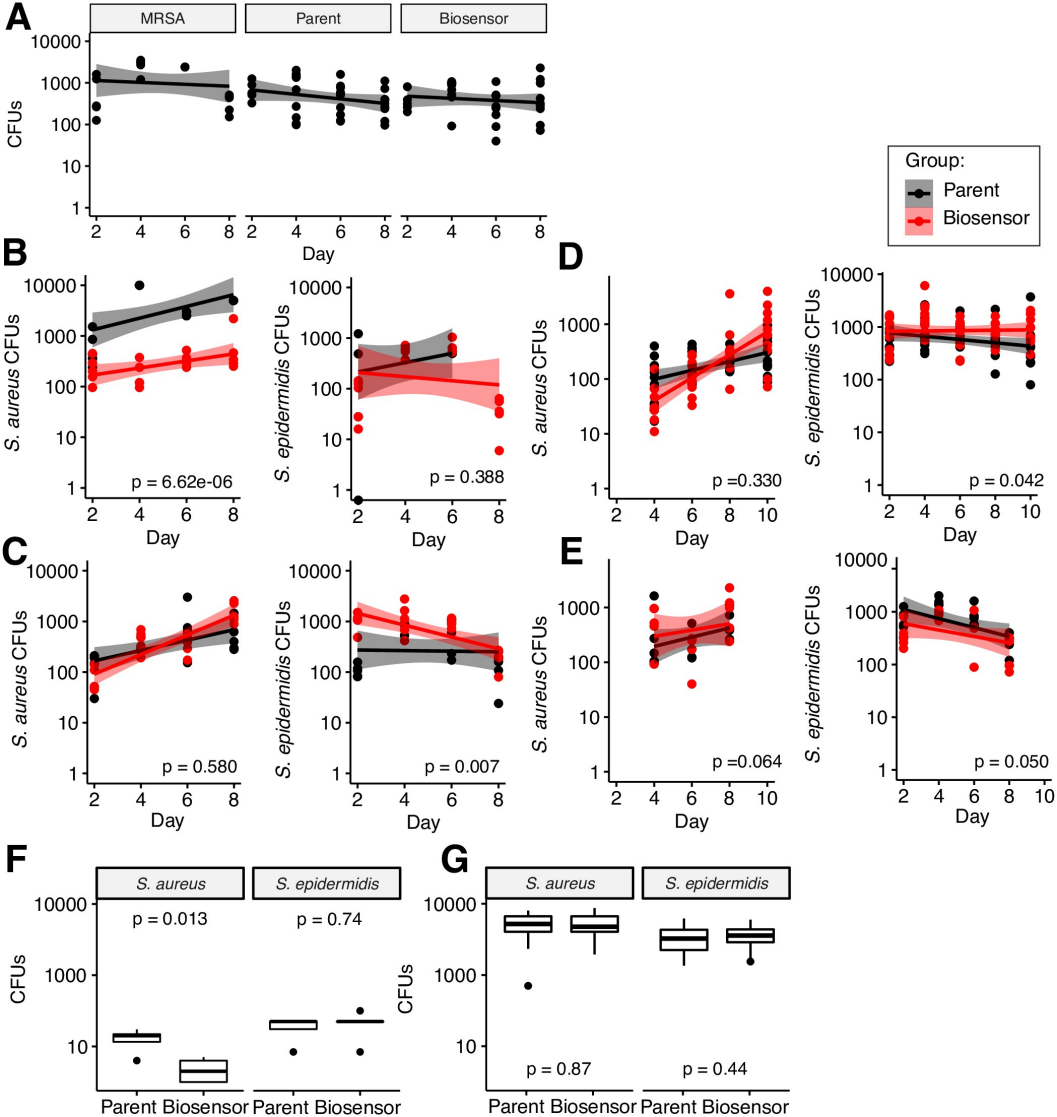
Trials to define biosensor control of MRSA proliferation *in vivo*. Germ-free C57BL6/J mice were colonized on the ears on days 0, 2 and 4, and growth of *S*. *epidermidis* (*agr* type I lysostaphin producing biosensor or Tü3298Δ*agr* parent strain) and *S*. *aureus* was quantified by swabbing the ear, directly spreading on mannitol salt agar, and then counting CFUs on days 2, 4, 6, 8, and 10 (select cases). In **A)** we show representative control samples for colonization alone by *S*. *aureus* USA300 (MRSA), *S*. *epidermidis* parent, or *S*. *epidermidis* biosensor strains. **B)** and **C)**, **D)** and **E)**, and **F)** and **G)** are replicate experiments. In **B)** and **C)**, we examined biosensor efficacy in suppressing growth of MRSA when co-colonized. MRSA was mixed with the biosensor or parent in a suspension at a 1:100 ratio (10^7^/10^9^ CFUs, respectively) and then applied to the mouse ears. n = 5 mice for all groups. Spearman linear regression line is shown with 95% confidence intervals. P-value to assess statistical significance in CFU counts between groups, accounting for time as a covariate, was determined within each panel by ANCOVA. In **D)** and **E)**, we examined biosensor efficacy in preventing colonization (and suppressing growth), colonizing mouse ears with biosensor or parent alone at day 0, then challenging with the suspension of *S*. *aureus* USA300 with *S*. *epidermidis* biosensor or parent on day 2. n = 5 for all groups. In **F)** and **G)**, we examined growth in a wound model, in which mice underwent a dorsal punch biopsy which was then inoculated with the suspension of *S*. *aureus* USA300 plus biosensor or parent. After 48 h, dorsal skin surrounding the wound was harvested for CFU quantitation on mannitol salt agar. n = 5 mice/group (left), n = 12/group (right). P-value was determined by bidirectional t-test.

We next sought to determine whether a staphylococcal biosensor could inhibit MRSA colonization or proliferation *in vivo* using a gnotobiotic C57BL6J mouse model. Mouse ears were treated with petroleum jelly suspensions of the lysostaphin-expressing type I biosensor strain, its parent strain Tü3298Δ*agr* as a negative control, type I *S*. *aureus* MRSA strain USA300, or a combination thereof. For all experiments (except where otherwise noted, see Fig 3 legend), inocula included 10^7^ CFU of MRSA and 10^9^ CFU of *S*. *epidermidis*. While these densities of bacteria are higher than estimated in human skin [38], they are typical of experiments applying human staphylococci to mouse skin [39]. Microbial mixtures were applied at 0, 2, and 4 days, and colonization of *S*. *epidermidis* or *S*. *aureus* was assayed by colony-forming unit (CFU) quantitation for up to 10 days. As a control, both *S*. *epidermidis* strains colonized adequately, consistent with our prior use of this model, and no significant fitness defect was noted in the biosensor compared to its parent strain (Fig 3A). We performed several different iterations and replicates of this experiment to model potential real-world situations, and to attempt to resolve high variance in the model. We altered: Fig 3B and 3C) dosage, Fig 3D and 3E) timing of colonization (concurrent colonization vs. pre-colonization by the biosensor), or Fig 3F–3G) changing the host environment by assessing colonization dynamics in breached skin (skin punch biopsy wound).

Biosensor efficacy would, ideally, manifest as low/no increase in *S*. *aureus* CFUs over the course of the experiment, compared to the parent strain (slope = 0 vs. slope > 0, or CFUs biosensor << CFUs parent at endpoint, respectively). Although conducted rigorously with germ-free mice, we found that there was 1) effective but very high variance in colonization (or quantitation) of either staphylococci, both within- and between- cohorts/cage. We used a range of birth cohorts of younger mice (~1 month of age), and randomized and individually housed mice where possible, but could not identify a consistent confounder that could explain the variance. 2) Interestingly, we observed that the parent strain alone could be effective at blocking *S*. *aureus* growth, suggesting that colonization resistance could, to a degree, be imparted by physical occupation of the environmental niche. 3) *S*. *epidermidis* colonization also varied, and tended to decrease over time, while *S*. *aureus* typically increased (with or without biosensor), suggesting that this strain of *S*. *epidermidis* may not be well suited to growth on the mouse skin, which we believe could impact efficacy of antimicrobial production. Taken together, the numerous variables with the colonization model resulted in 4) variable biosensor efficacy from cohort to cohort, which we believe was a consequence of insufficient density-dependent production of *S*. *aureus* AIP, insufficient *S*. *epidermidis* cells to produce adequate antimicrobial to significantly reduce *S*. *aureus* growth, and/or environmental factors that otherwise affected *S*. *epidermidis* fitness and antimicrobial production. The gnotobiotic model (similar to results in an *in vitro* 3D reconstructed human epidermis model), suffered from high variance in colonization efficacy, which we believe impacts the underlying biology of our biosensor, which requires sufficient accumulation of *S*. *aureus* AIP for antimicrobial production.

## Discussion

While far from comprehensive, these results provide a proof-of-concept that *S*. *epidermidis* can be engineered to detect and respond to *S*. *aureus*, express exogenous antimicrobial proteins or peptides under this system, and potentially control MRSA proliferation via these mechanisms. A similar *S*. *aureus* sensor mechanism was recently developed by Lubkowicz *et al*. in *Lactobacillus reuteri*, showing the impressive molar sensitivity of type I *agrCA* [40]. We conjectured that our use of *S*. *epidermidis*, which unlike lactobacilli, ubiquitously colonizes every human skin site [27], and placing antimicrobial genes as an output, would open the door to using this technology not just as a detector but also a potential prophylactic or therapeutic in the skin. While antimicrobials could be expressed constitutively in *S*. *epidermidis*, we also sought to circumvent potential fitness and auto-toxicity burdens to the host and potentially adverse disruptions to the native microbiota.

However, we found that while our biosensor performed impressively and reproducibly *in vitro*, *in vivo* efficacy was highly variable. We attribute this to numerous potential factors previously discussed, most likely, in our opinion, differential colonization efficacy, density, and ratio of *S*. *epidermidis*:*S*. *aureus*, as proximity is requisite for biosensor activation. We note that we were unable to ascertain density of staphylococcal colonization, or actual amount of AIP present that would be sufficient to activate the biosensor. In addition, we acknowledge that we performed only qualitative assessments of antimicrobial production, as we deemed *in vivo* efficacy to be the first bar to justify a rigorous quantitation as a function of colonization efficacy, density, and environmental conditions. While imaging or mass spectrometry presumably could be used to prove density as a variable, due to the longitudinal nature of the experiment and the difficulties in accurate inference, we did not attempt this. From experiments in reconstructed human epidermis, we have seen ‘patchiness’ in colonization with GFP-labeled strains that supports this hypothesis. In addition, recovery and quantitation by swabbing may impart additional variance given potential grooming by the animals or adherence of cells to the skin.

Similarly, antimicrobial activity is likely highly localized to *S*. *epidermidis* cells, as supported by our *in vitro* data. An additional control for future *in vivo* experiments might include a strain that constitutively produces the antimicrobial of interest (or our inducible version from 2C), to specifically evaluate the ability of the biosensor to sense then produce antimicrobial. In addition, we note that even when a trial could be deemed successful, MRSA was never entirely cleared. This may be an inherent disadvantage of deriving the biosensing mechanism from a quorum-sensing regulator, as these have evolved for population density-dependent signaling, rather than maximum sensitivity. A potential future direction for this technology could be to optimize the sensitivity of the AgrCA regulator with directed evolution. Higher sensitivity may facilitate a greater reduction in MRSA colonization, a more rapid suppression of its expansion, or importantly, given our *in vivo* results, a greater tolerance to low relative density of the biosensor with respect to its target. In addition, screening/engineering additional promoters for higher production of the antimicrobial may improve efficacy. Finally, other *agr* type biosensors should be characterized, in addition to other *S*. *epidermidis* strains, which may have different colonization dynamics. We pursued the type I biosensor as the most likely to be clinically pertinent, given the prevalence and virulence of *agr* type I strains [28]. As an aside, we extensively attempted to engineer an omnisensor (one cell capable of sensing all 4 types), using different regions of the *agrA*, *agrC*, and P2/P3 from all *agr* types. However, these efforts were not fruitful, likely because of high homology between the regions, despite introducing numerous synonymous base pair changes.

While we were encouraged by the strong *in vitro* performance of this “detect and destroy” probiotic approach, we feel that our inconclusive *in vivo* results reflect real-world challenges facing this and other topical probiotics. We and others established the biodiversity, interindividual, and intraindividual differences in the skin microbiome [13, 27, 41, 42]. Colonization of exogenous *S*. *epidermidis* may be hampered by numerous physiological considerations, including penetrance through the stratum corneum and colonization into deeper adnexal structures, differing physiological conditions of different skin sites (e.g., oiliness/moistness/dryness/pH), and/or competition against the endogenous microbiota, including other *S*. *epidermidis* strains which may have pre-adapted to the niches of various skin sites. In addition, it is possible that *agr* alters colonization efficacy, and we used an *agr* deficient *S*. *epidermidis* strain–reports on *agr*’s requirement for colonization short and long-term are conflicting, or have been assessed in different species [43–45]. Additional characterizations of *S*. *epidermidis* and *S*. *aureus* growth dynamics and interactions *in vivo* may be warranted for future applications to create an improved model that can account for these real-world differences.

Finally, an important consideration for genetically engineered microbial therapeutics are methods of biocontainment, preventing the transmission of the heterologous organism from the target site or from person to person, e.g., auxotrophy, foreign nucleotides and amino acid usage, and kill switches have been proposed or utilized [46, 47]. While understanding the underlying biology of colonization of exogenous strains will be a critical next step, we hope that this work provides an example of a potential initial design of a “detect and destroy” probiotic approach for mitigation of *S*. *aureus* infection.

## Supporting information

S1 File Raw data underlying figures. (XLSX)
